# Assessing diversity of King Crab *Lithodes* spp. in the south‐eastern pacific using phylogeny and molecular species delimitation methods

**DOI:** 10.1002/ece3.9143

**Published:** 2022-07-31

**Authors:** Ramona Pinochet, Luis Miguel Pardo, Leyla Cárdenas

**Affiliations:** ^1^ Programa de Doctorado en Biología Marina, Facultad de Ciencias Universidad Austral de Chile Valdivia Chile; ^2^ Centro de Investigación de Dinámica de Ecosistemas Marinos de Altas Latitudes (IDEAL) Valdivia Chile; ^3^ Instituto de Ciencias Marinas y Limnológicas, Facultad de Ciencias Universidad Austral de Chile Valdivia Chile; ^4^ Instituto de Ciencias ambientales y evolutivas, Facultad de Ciencias Universidad Austral de Chile Valdivia Chile

**Keywords:** genetic diversity, *Lithodes* diversity, mitochondrial lineages, molecular delimitation methods, south‐eastern Pacific

## Abstract

The purpose of this study was to test the hypothesis that the genetic diversity of commercially significant species of King Crabs (*Lithodes* spp.) along the south‐eastern Pacific (SEP) comprises different independent evolutionary units (IEUs) with spatially isolated distribution. Nine localities from inner and open waters along the SEP Chilean coast (39°S‐55°S) were sampled. We analyzed sequences from 173 individuals for the mitochondrial gene Cytochrome oxidase I (COX‐I), 151 individuals for the Internal Transcribed Spacer 1 (ITS) and 135 for the structural ribosomal RNA (28S). Genetic delimitation was performed through three analytical methods: ABGD, GMYC, and its Bayesian implementation, bGMYC. Bayesian phylogenetic analyses and haplotype networks were also performed. Divergence time between clades was assessed for the COX‐I marker and estimated from known evolutionary rates for this marker in other crustacean species and fossil calibration from other Anomuran species. Delimitation analyses, phylogenetic analyses, and mitochondrial haplotype networks suggested the presence of two deeply divergent mitochondrial lineages of *Lithodes* in the SEP, referred to as Clade1 and Clade 2. Nuclear markers showed low phylogenetic resolution and therefore were unsuitable for molecular species delimitation. Divergence time analysis of the mitochondrial lineages suggests a separation between Clades of approximately 2.3 Mya. The divergence time obtained suggested that Pliocene glaciations and deglaciations cycles could be involved in hybridization events between *Lithodes* IEUs at southern tip of South American coasts. The different frequencies of *Lithodes* haplotypes in inner and open water environments along SEP coasts could be explained by events such as the last glacial maximum or by differences in the adaptation of each clade to different environments. These findings support the necessity of evaluating the taxonomic status of *Lithodes* individuals found along SEP coasts under an integrative taxonomy approach or through markers with other evolution rates than those already used.

## Graphical abstract

The purpose of this study was to assess the biodiversity of individual Lithodes spp. across the Southerneast Pacific with multilocus molecular markers. A complex of independent evolutionary units comprising at least two genetically distinct lineages with different spatial distribution is observed.

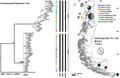

## 1 INTRODUCTION

The importance of biodiversity and distribution of species surveys lies in the fact that these factors are the basic units for sustainable management and biodiversity conservation (King, 2007). This involves the identification and delimitation of species; from there, patterns of genetic subdivision of the species in the geographical space, which are crucial to understanding events at the population level, can be studied (Carvalho & Hauser, 1995; Carvalho & Nigmatullin, 1998). In the case of exploited resources, this information is of great interest to fishery managers since populations are the biological units on which management and conservation strategies are applied (Carvalho & Hauser, 1995; Waples et al., 2008). Therefore, knowledge of the diversity and distribution of species targeted by the fishery can improve fishery management plans by better defining their operational units.

However, species delimitation can be a delicate task as not all characteristics will vary at the same time and not all criteria and methods used to assess these characteristics will result in the same species delimitation (De Queiroz, 2007). Due to the large number and incompatibility of some species concepts (De Queiroz, 2007; Mayden, 1997), a good option would be to refer to independent evolutionary units (IEUs, Jones, 2017; Lim et al., 2012) instead of species, especially if only one or a few concepts are considered to define them.

Establishing the diversity and distribution of IEUs for cryptic organisms is especially challenging when the morphological characteristics are not sufficiently divergent to delimit between IEUs (e.g., Bickford et al., 2007; Lefébure et al., 2006). In some Anomura crustacean IEU complexes, morphological characteristics such as coloration, cephalothorax shape, and carapace spinulation patterns (size, number, and distribution) have not been taxonomically reliable characteristics in delimiting cryptic IEUs of porcellanids (Negri et al., 2014; Werding & Hiller, 2017), munids (Machordom & Macpherson, 2004), and lithodids (Pérez‐Barros et al., 2015). In such cases, since morphological differences between cryptic individuals are minimal, other traits may be used to delimit IEUs; these include behavior (Crossley, 1986), karyotype structure (Amaro et al., 2012), protein composition (Fong & Garthwaite, 1994), DNA sequences (Hebert et al., 2004), or even geometric morphometrics traits (Francuski et al., 2011).

**FIGURE 1:**
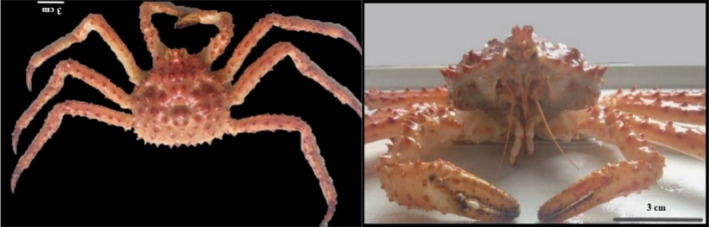
Dorsal (left) and frontal (right) view of a male specimen of the morphospecies *Lithodes santolla* Molina 1782. Photographs taken during sampling in Tenaun, Chile, 2018.

The delimitation of IEUs, which lies at the core of taxonomy and ecology, is related to the boundary between micro‐ and macro‐evolution and determines number and boundaries of IUEs (De Queiroz, 2007). By analyzing DNA sequences, IEUs can be identified as “separately evolving metapopulation lineages” (sensu De Queiroz, 2007). Recent developments in analytical methods in phylogenetics, namely, relationships among lineages and the membership of individuals within these groups, can now be evaluated including the generalized mixed Yule‐coalescent (GMYC) model (Pons et al., 2006), its Bayesian implementation (bGMYC) (Reid & Carstens, 2012), and the Automatic Barcoding Gap Discovery (ABGD) model (Puillandre et al., 2012), among others. Furthermore, the delimitation of IEUs based on only one type of genetic marker and one type of approach could be inconclusive, and the use of a multilocus analysis including different statistical approaches is thus highly recommended so as to obtain more precise and accurate results (Carstens et al., 2013; Dudgeon et al., 2012; Moore, 1995; Reid & Carstens, 2012). Southern King Crab (SKC) fishery is recognized as an activity of high social and economic importance in Chile (Bozzeda et al., 2019; Molinet et al., 2020; Nahuelhual et al., 2018). Between 2016 and 2019, this fishery reached landing levels of 5179 mean tons annually (Servicio Nacional de Pesca y Acuicultura, SERNAPESCA, 2020); the haul is primarily destined for international markets, primarily China (Enexpro project, 2017). Until 2018, this fishery registered 7115 artisanal fishers (Subsecretaría de Pesca y Acuicultura, SUBPESCA, 2018) while many other workers are employed in the related industrial and commercial areas. The SKC fishery operates along the SEP coast from Valdivia (39°S) to Cape Horn (56°S), including channels, fjords, gulfs (inner waters areas), and also areas offshore (open waters). The fishery range coincides with the range described for SKC individuals along the SEP coast (Bozzeda et al., 2019; Molinet et al., 2020; Retamal & Moyano, 2010). Despite of the importance of this fishery, studies that describe the biodiversity and distribution of *Lithodes* IEUs in this area are scarce (e.g., Retamal, 2012). Currently, based on classical morphological characteristics, the SKC constitutes a single species, *Lithodes santolla* Molina, 1782 (Crustacea: Anomura, Figure 1) distributed along the SEP coast (e.g., Lovrich, 1997; Sierpe & Sanhueza, 2003) co‐habiting with *Lithodes turkayi* Macpherson, 1988 and *Lithodes confundens* Macpherson, 1988 at the southern tip of South America (Boschi & Gavio, 2005; Lovrich, 2014; Lovrich & Tapella, 2014). In a recent phylogenetic analysis with two mitochondrial markers 16S and COX‐I, Pérez‐Barros et al. (2015) analyzed two morphospecies of *Lithodes* from the Atlantic and Pacific coasts (*L. santolla* and *L. confundens*) and concluded that morphological delimitation was incongruent with the genetic delimitation. They recognized three morphological clusters with different number of spines each and two mitochondrial genetic lineages. On one hand, they evidenced inter‐lineage crypticism, with overlapping morphs, and on the other hand, one of the *Lithodes* genetic lineages (clade 1) showed greater morphological variation with recognition of three distinct morphs (that can occur as a result of an adaptative or plastic response to the diversity of habitats populated by SKC individuals). These authors are the first ever to suggest the potential existence of a complex pattern of genetic lineages in *Lithodes* from SEP coasts.

Given the importance of clarifying the biodiversity composition and the spatial distribution of *Lithodes* throughout the SEP for both conservation and fishery management issues, in this study we aim to delimitate IEUs of *Lithodes* through an exhaustive sampling collection along the SEP coast. Three molecular genetic markers, one mitochondrial and two nuclear, and four different approaches are considered for the genetic delimitation analysis. We hypothesize that IEUs of *Lithodes* along the SEP coast are constituted by at least two genetically distinct lineages with different spatial distribution.

## 2 METHODS

### 2.1 Sample collection, DNA isolation, amplification, and sequencing

**FIGURE 2:**
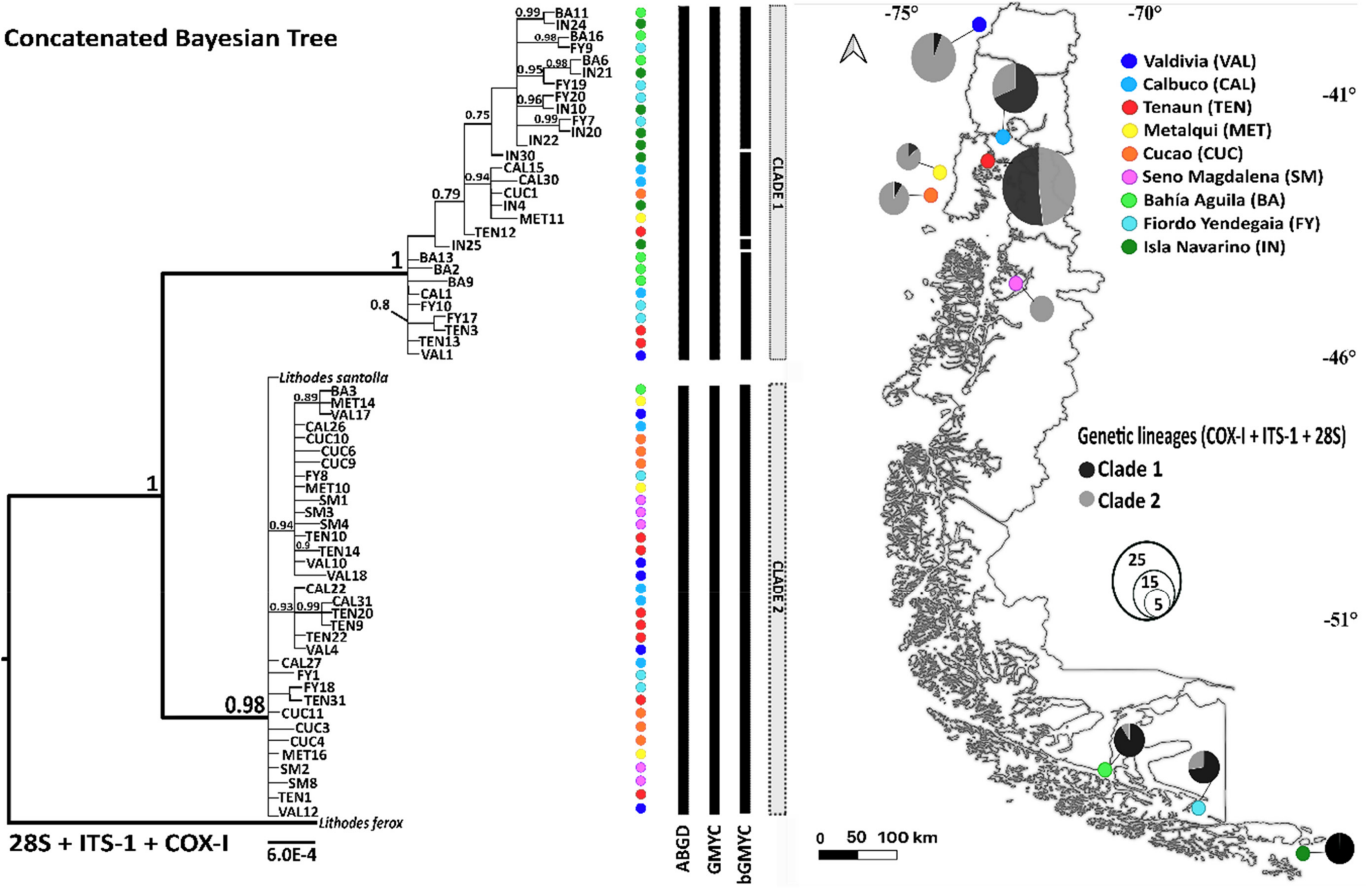
Left side: Bayesian phylogenetic reconstruction of haplotypes obtained with MrBayes showing the main clades. Values above branches indicate the BY posterior probabilities (above 0.7). Circles assigned to each individual in the Bayesian tree represent its locality of origin depicted in the map. Center: summary of IEUs delimitation of *Lithodes* individuals along the SEP coast. From left to right, black bars represent species delimitation obtained from ABGD, GMYC and bGMYC methods. Right side: map showing frequencies of each Clade classified according to the phylogeny of concatenated genes in each sampled location. Pie charts' sizes are proportional to the number of individuals collected at each sampling location.

Individuals of the morphospecies *Lithodes santolla* Molina 1782 were collected from 39°S to 55°S, from nine localities (Figure 2, right side). Four sampling locations were located in open waters areas: Valdivia (39°48'S;73°14'O), Metalqui (42°12'S; 74°22'O), Cucao (42°43'S; 74°47'O), and Isla Navarino (55°28'S; 66°52'O), and five were in inner water areas: Calbuco (41°47'S; 73°7'O), Tenaún (42°20'S; 73°22'O), Seno Magdalena (44°37'S; 72°57'O), Bahía Águila (44°37'S; 72°57'O), and Fiordo Yendegaia (54°51'S; 68°47'O). Individuals were collected by commercial vessels and by SCUBA diving.

Samples were taken from the muscle tissue of one periopod of each individual or, in the case of smaller individuals, the entire individual and were preserved in ethanol (90%) for later DNA extraction. The DNA was extracted according to the “solid tissue protocol” described in the Quick‐DNA Miniprep Plus Kit (Zymo Research; Irvine, CA, USA). We considered three molecular markers with different evolutionary rate, one mitochondrial marker, the Cytochrome Oxidase I (COX‐I), and two nuclear markers, the Internal Transcribed Spacer 1 (ITS‐1) and the structural ribosomal RNA 28S (28 S) for the large subunit (LSU). The universal primers defined by Folmer et al. (1994) were used to amplify the COX‐I. The primers defined by Chu et al. (2001) were used for the ITS‐1. These genetic markers were selected as they have been previously used for delimitating *Lithodes* species (e.g., Hall & Thatje, 2018; Noever & Glenner, 2018) and for their availability in public database GENBANK. The primers to amplify the gene 28S were designed in the Geneious version 11.1.5 (Kearse et al., 2012) based on sequences of the 28S gene from *Lithodes* individuals available in the public database GenBank (KF182602, HM020859, HM020861, HM020855, FJ462642, AY596100) (Benson et al., 2014) (for more information about primers, see TableS1.1). For all molecular markers, polymerase chain reactions (PCR) were performed on a thermal cycler (T100 Biorad Labs) in a final volume of 30 μl and a mixture as follows: 1 μl of DNA (50 ng/μl); 1X Taq buffer+KCl; 2.5 μM of dNTPs; 5 U/μl of Taq DNA polymerase (Fermentas, Thermo Scientific); 10 μM of each primers; 1X BSA (100X) (New England, Biolabs) and 25 mM MgCl2. Alignment temperature varied over thermal cycles according to marker and even between individuals from different localities. The quality and quantity of PCR fragments were verified through electrophoresis in agarose gels (1.5%, RedGel staining) and sequenced in both directions with an ABI3730 x 1 Automatic Sequencer at Macrogen Inc. (Seoul, South Korea). The sequences were edited with Geneious version 11.1.5 (Kearse et al., 2012). Multiple alignments of COX‐I, ITS‐1, and 28S with Geneious alignment algorithm (Cost matrix = 65% similarity; Gap open penalty = 12; Gap extension penalty = 3) and the concatenation of genes were performed within Geneious prime 2019.0.4 (https://www.geneious.com).

### 2.2 Phylogenetic analyses

Phylogenetic reconstructions for each molecular marker and for the concatenated data were conducted under Bayesian inference (BI) with MrBayes 3.2.7 software available on the CIPRES online platform (Miller et al., 2010). Substitution models for each marker were obtained with MEGA v10.2.5 (Kumar et al., 2018) and selected based on the Akaike Information Criterion (AIC) and CIPRES platform options: JC (Jukes & Cantor, 1969) for 28S, JC + I for ITS1 and HKY + G for COX‐I. In Bayesian analysis with concatenated dataset, we set specific substitution models for each marker. Monte Carlo Markov Chains (MCMC) were carried out with 10 million generations, with samples of phylogenetic trees taken every 1000 generations. We discarded the first 25% of trees as “burn‐in” and the remaining trees were used to generate a majority‐rule consensus tree. Temperature parameter (final Temp value) was maintained to default value of 0.5. To reveal the genealogical relationship among the mitochondrial haplotypes, networks were generated using HapView (Salzburger et al., 2011).

### 2.3 Molecular delimitation

IEUs delimitation analysis considered the concatenated dataset and three different approaches. The ABGD (Puillandre et al., 2012) does not require a prior phylogenetic tree and based on genetic distance values, automatically finds the location of a barcode gap between candidate species or between intraspecific and interspecific diversity. The ABGD analysis was run using the web version (http://www.abi.snv.jussIEUs.fr/public/abgd/abgdweb.html) with the best substitution model obtained for the concatenated dataset in MEGA v10.2.5 (Kumar et al., 2018), Kimura's two‐parameter model (K2P model), and with priors that ranged from Pmin = 0.001 to Pmax = 0.1 with 10 steps. Next, the GMYC (Pons et al., 2006) single threshold model was carried out using the Species Limits by Threshold Statistics approach in the Splits and Ape packages of R program v. 4.0.5 (www.r‐project.org). This method is based on the approach that delimits species by adjusting ramified models of intra and inter species to a reconstructed gene tree (Reid & Carstens, 2012). Finally, the bGMYC was also carried out in R program v 4.0.5 with the bGMYC package (Reid & Carstens, 2012). This Bayesian analysis was based on samples from the posterior distribution of gene trees, thus allowing uncertainty in both the topology and branch lengths to be reflected in posterior parameter estimates (Reid & Carstens, 2012). A range of probabilities >0.95 was considered as strong evidence that the groups compared were conspecific, while a range of probabilities <0.05 strongly suggested that the groups compared were not conspecific (Reid & Carstens, 2012). Inter‐lineage genetic distance for COX‐I dataset was calculated using Kimura's two‐parameter model (K2P model) in MEGA v10.2.5 (Kumar et al., 2018).

### 2.4 Estimation of divergence time

Divergence times (i.e., time to the most recent common ancestor, TMRCA) for nodes of interest in the phylogeny among mitochondrial haplotypes were estimated using a Bayesian approach with BEAUti & BEAST v2.6.2 (Bouckaert et al., 2019). We implemented a relaxed molecular clock with lognormal distribution (Drummond et al., 2006) to mtDNA sequences using a substitution model of HKY + G obtained with MEGA v10.2.5 (Kumar et al., 2018) and an uncorrelated‐lognormal (ucln) model of molecular evolutionary rate. A birth‐death speciation prior was used to estimate branching rates in the phylogeny. Taking into account published substitution rates for COX‐I in other crabs (Ketmaier et al., 2003; Schubart et al., 1998; Sotelo et al., 2009; Xu et al., 2009), we set the clock rate to 1.0E‐8. Ucld.mean prior was set to gamma distribution with an initial value of 1.0E‐8, scale of 1000 and Shape of 0.001. We included one calibration point previously estimated for the tmrca of Lithodidae+Hapalogastridae of 18 Mya (Bracken‐Grissom et al., 2013) and two fossil calibrations for Paguridae of 30 Mya (*Pagurus malloryi*, Schweitzer & Feldmann, 2001) and for Paralomis of 15 Mya (*Paralomis debodeorum* Feldmann, 1998). Accession numbers for the COX‐I sequences of other Anomura species used in this analysis are available in Table S1.2. Clade 1 was represented by a sample of Fiordo Yendegaia (FY12) and Clade 2 by a sample of Valdivia (VAL25). We implemented one run in BEAST with an MCMC chain length of 50 million generations and trees sampled every 1000 generations. ESS values of each parameter (ESSs > 500) and convergence of the stationary distribution were checked using the software Tracer v1.7.1 (Rambaut et al., 2018).

## 3 RESULTS

The number of total sequences obtained for each genetic molecular marker are detailed in Table 1. Analysis of our sequences alignments revealed 40 variable sites (38 in COX‐I and two in ITS‐1), of which 37 were parsimony informative (35 in COX‐I and two in ITS‐1). In ITS‐1, the two informative sites correspond to substitutions. Alignment lengths for each genetic marker are specified in Table S1.1. The sequences were deposited in GenBank (Accession number for COX‐I: ON807360‐ON807532; 28‐S: ON868924‐ON869058; ITS‐1: ON869067‐ON869217).

**TABLE 1:** Sampling localities, coordinates and number of sequences obtained for each genetic marker and for the concatenated dataset by locality.

Locality	Coordinates	Number of Sequences			
		COX‐I	ITS‐1	28‐S	Concatenate
Valdivia	39°48′00″S‐ 73°14′00″O	28	17	21	16
Calbuco	41°47′47″S‐ 73° 7′48″O	31	25	17	16
Tenaun	42°20′2″S‐ 73°22′59″O	49	33	29	27
Metalqui	42°12′9″S‐74°22′31″O	13	14	8	7
Cucao	42°43′37″S‐74°47′20″O	11	12	12	11
Seno Magdalena	44°37′23″S. 72°57′25″O	6	8	8	6
Bahía Águila	53°47′11″S‐ 70°58′26″O	13	13	15	11
Fiordo Yendegaia	54°51′3″S‐ 68°47′25″O	15	15	14	11
Isla Navarino	55°28′16″S‐ 66°52′50″O	13	14	12	9
Total		173	151	135	114

### 3.1 Phylogenetic analysis

The number of sequences for each genetic molecular marker are detailed in Table 1. Alignment lengths for each genetic marker are specified in TableS1.1. Analysis of the alignments with own sequences revealed 40 variable sites (38 in COX‐I and two in ITS‐1), of which 37 were parsimony informative (35 in COX‐I and two in ITS‐1). Concatenated 28S, ITS‐1, and COX‐I alignments resulted in 1618 bp length sequences. These concatenated datasets include a total of 116 sequences, with 114 own sequences, a sequence of *L. ferox* used as the outgroup and a sequence of *L. santolla* used as the reference for our ingroup. Both *L. ferox* and *L. santolla* sequences were obtained from concatenation of sequences available for the three markers in GenBank (HM020856, HM021009, KY426276, KF182602, HM021015, KM887467).

The phylogenetic tree of the COX‐I gene showed the divergence of two main clades supported by the maximum probability value (PP = 1). The ITS‐1 marker phylogeny showed a single monophyletic clade supported by a high probability value (PP = 0.9) constituted by a few individuals, and the remaining individuals formed a polytomy (Figure S1.3 and S1.4). The 28S marker phylogeny showed all individuals forming a polytomy with the outgroup (*Lithodes ferox*, GENBANK accession number HM020856).

The phylogenetic analysis of *Lithodes* individuals using concatenated genetic data showed a monophyletic group that included all the samples from our study (posterior probability, PP = 1) (Figure 2). Within this group, two statistically well‐supported subclades were revealed (PP = 1 and 0.98 for Clades 1 and 2, respectively). Clade 1 consisted of 54 *Lithodes* individuals from all sampled sites. Clade 2 consisted of 61 individuals from eight sampled locations, that is, all locations except Isla Navarino. The length of the branches suggests that Clade 1 would be more divergent than Clade 2.

The phylogeny evidence co‐occurrence of both clades among almost all locations. However, spatial trends in the frequencies of Clade 1 and 2 were noted (Figure 2). Individuals collected in the southernmost localities belong almost exclusively to Clade 1 whereas individuals from northern Patagonia belong mostly to Clade 2. In northern Patagonia, the frequency of each clade was similar in inner water localities, but in open water localities, Clade 2 was found in greater frequency.

**FIGURE 3:**
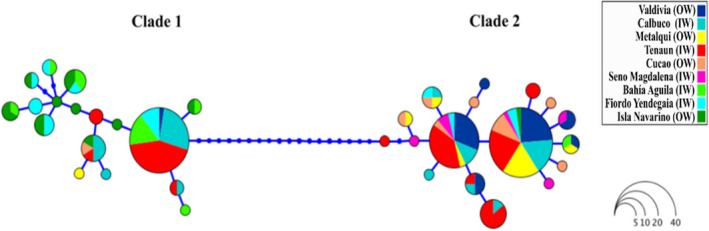
Haplotype network based on COX‐I gene for 174 individuals of *Lithodes* sp. Circle sizes are relative to haplotype frequencies. Colors represent each locality. OW: Open waters or IW: Interior waters denote environment of origin.

A haplotype network based on COX‐I showed a 20‐step mutational separation between the mitochondrial Clades 1 and 2 with 17 haplotypes identified within each clade (Figure 3). Interestingly, the most common haplotypes within each Clade were shared by individuals from different locations. The most common haplotype in Clade 1 is shared almost entirely by individuals from inner water locations (Teknion, Calbuco, Bahía Águila, and Fiordo Yendegaia). Clade 2 has two frequent haplotypes in the center of the network containing individuals from all sampled locations.

### 3.2 Molecular IEUs delimitation

The ABGD analysis (Figure 2) showed a clear threshold partitioning in the dataset in the frequency histogram of genetic distances where intra‐ and inter‐specific distances do not overlap. Initial partition of the sequence data at each value of the prior intraspecific divergence (P) (0.001–0.0215) divided data in three potential genetic clusters; one corresponding to Clade 1, the other to Clade 2 (as defined in the BY analysis) and the last one to the outgroup. Intraspecific sequence divergences ranged from 0.1 to 1.29%. The GMYC (Figure 2) yielded two putative genetic clusters with a significance value of 5.6e–08 and a threshold time of −0.0007. The single threshold approach proved to be accurate for our dataset since the multi‐threshold approach recognized 68 genetic entities, far overestimating number of putative IEUs. The analysis using bGMYC (Figure 2) yielded five putative genetic clusters, partitioning Clade 1 into four groups. The bGMYC resulted in a significant delimitation of Clades 1 and 2 as distinct genetic clusters, with most pairwise comparisons having a non‐conspecificity probability (*p* ≤ .05). The mean genetic distance between *Lithodes* mitochondrial clades recovered was 4.58% (SD = 0.92).

### 3.3 Divergence time analysis

**FIGURE 4:**
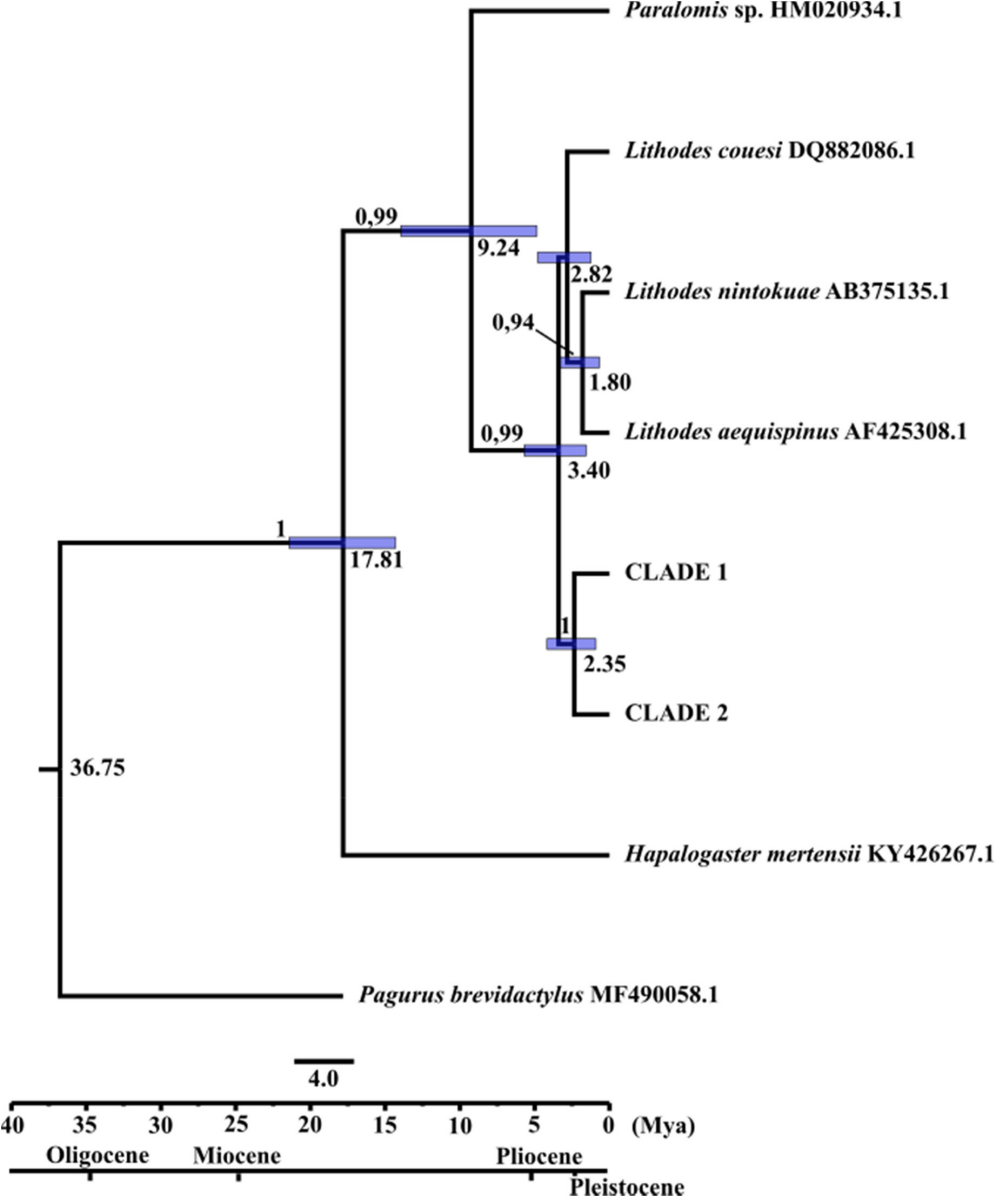
Divergence time chronogram based on COX‐I using Bayesian evolutionary analysis via sampling trees using BEAST with estimated and fossil calibrations. Values above branches indicate the BY (Bayes) posterior probabilities (above 0.7). Divergence time estimate (Mya) between taxas are indicated in each node. Shaded bars indicate 95% highest posterior density intervals. Geological epochs are shown in concordance with the timeline in millions of years (Mya).

The divergence time analysis showed that the two *Lithodes* lineages would have diverged at around 2.3 Mya (95% highest posterior density HPD95: 0.914–4.177 Mya), suggesting a split during the Pliocene epoch (5.3–1.8 My) (Figure 4).

## 4 DISCUSSION

Delimitation approaches tested here based on three different genealogical analytical methods (ABGD, GMYC, and bGMYC) showed similar results and consistently identify two genetic clusters of *Lithodes* in SEP coasts. Inter‐lineages divergence values obtained with concatenated data using the COX‐I gene were similar from others found among some crustacean species (e.g., Meyer et al., 2013; Naim et al., 2020). Phylogenetic and lineages delimitation analyses were consistent and revealed a high divergence between the two clades (Clade 1 and Clade 2). It is worth mentioning that COX‐I mitochondrial marker was the main determinant of the divergence observed between clades. In this sense, the present results are coherent with those from Pérez‐Barros et al. (2015), with the presence of two sympatric mitochondrial lineages of *Lithodes* inhabiting SEP coasts. Nuclear markers did not show strong phylogenetic resolution in the individual phylogenies for each marker nor in the analysis with the concatenated dataset. This suggests that the nuclear markers here used may not be variable enough and new molecular diagnostic approach using a wide genomic screening perspective (like SNPs) could be the next step to clarify this point (Dufresnes et al., 2021; Herrera & Shank, 2016; Ortiz et al., 2021).

According to divergence time estimates, the evolutionary history of *Lithodes* mitochondrial lineages would have been affected by climatic fluctuations during Pliocene‐Pleistocene glaciations and deglaciation cycles. Late Pliocene and early Pleistocene was an epoch of global cooling and repeated glacial periods with deep ocean temperature bordering 0°C (Hansen et al., 2013). A cooling period is registered around 2.8–2.5 Mya and 2.0–1.7 Mya (Dupont & Leroy, 1999; Marlow et al., 2000). Moreover, late Pliocene was also the time of surface cooling, intensification of the frontal gradients, northward movements of the Polar Front Zone in the Southern Ocean and increase in vertical ocean stratification at high polar latitudes (Cortese et al., 2004; Diekmann et al., 2003; Hodell & Venz, 1992; Sigman et al., 2004; Warnke et al., 1992). These events and posterior glaciation and deglaciation cycles in Pleistocene epoch could have promoted hybridization events between *Lithodes* IEUs from each side of South America through primary contacts and secondary contacts at the southern tip of South American coasts.

Divergence time estimates are in agreement with previous studies on species of Anomura (Bracken‐Grissom et al., 2013). Furthermore, our results agree with those obtained by Pérez‐Barros et al. (2015), who estimated a divergence time between Clades 1 and 2 of *Lithodes* of about 1.2 Mya (our estimate had an HPD95: 0.914–4.177 Mya which includes 1.2 Mya).

Although individuals from both clades were found in almost all of the sampled locations, we observed differences in mitochondrial haplotype frequencies throughout the studied areas. Taking into account the date of divergence between *Lithodes* mitochondrial lineages, we hypothesized that their evolutionary history could be marked by constant changes in the population sizes and their distribution ranges. Along with other past events, the Last Glacial Maximum (LGM; approximately 18,000–20,000 years ago; Denton et al., 2010) may also have influenced the actual observed lineage frequencies in the Pacific coast. During LGM, several marine species contracted their distribution ranges due to the formation of an ice sheet; they survived in refuge areas and later recolonized surrounding areas after the retreat of the ice sheet and the increase in temperature after the LGM (e.g., Fraser et al., 2012; González‐Wevar et al., 2012; González‐Wevar et al., 2013; Montecinos et al., 2012). Clade 1 likely retreated southward during the LGM and then recolonized northern locations through channels and fjords. Likewise, the distribution of Clade 2 in northwestern Patagonia was restricted to open water areas, and they then recolonized inner waters and expanded southward. After the ice melted, the expanded ranges of both clades may have produced a more recent secondary contact, thus promoting contemporary hybridization and gene flow between clades. Another explanation is that the frequency distribution pattern of both clades in Pacific Ocean could be consequence of the historical biogeography and the different oceanographic regimens of the fjords and channels as consequences of the glaciations events and the river influences in the inshore Pacific areas. Indeed, the coastal ice sheet extended from 41°S to Cape Horn on the Pacific coast, over 1800 km, whereas the Atlantic coast was completely ice free (Rostami et al., 2000).

Along these lines, it would be interesting to conduct studies on habitat preference and/or physiological requirements of each *Lithodes* lineage related to different environmental factors. More analysis, including an exhaustive sampling collection in more locations and new molecular markers or wider genome‐scan approaches would allow us to detect footprints of selection with the aim of differentiating the global effects of the demographic evolutionary forces (e.g., gene flow, inbreeding, and genetic drift) from the local effects of selection (Balding & Nichols, 1995; Vitalis et al., 2001).

A serious limitation to the utility of molecular markers as a practical resource for species diagnosis is the human error and uncertainty in creating and curating reference libraries (Collins & Cruickshank, 2013). One of the complexities in our analysis is that the sequences for *L. santolla* and *L. confundens* available in the GenBank database were formerly named based on the morphology and sampling location, which could lead to misunderstandings when trying to clarify the taxonomic status of cryptic lineages of *Lithodes* with sympatric ranges. A clear example of this can be seen in the COX‐I phylogenetic tree, where some sequences recognized based on their morphology as *L. santolla* (KM887497, KM8874901, KM887487, KM887450, KM887469, KM887494, KM887495) show high homology with *L. confundens* sequences. From our COX‐I dataset, some individuals from Clade 2 showed high homology with *L. santolla* sequences available in the public Database GenBank (KM887460, KM887467, KM887492, HM020897, HM020902), and some sequences from Clade 1 showed high homology with *L. confundens* sequences (HM020900, HM020901, KC196536, KC196538, KM887493, KC196535, KC196537, KM887440, KM887441). According to the explanation provided above, this homology found should be treated with care, meaning that the clades do not necessarily represent genetic lineages of *L. confundens* and *L. santolla*. The classification of available sequences in the public database was done based on morphological characters. In many cases delimitations rely on subjective interpretations of morphological and/or DNA data.

In addition to the above, one of the main issues in the *Lithodes* species delimitation is the availability of curated sequences for other potential related species. For that, we choose the most popular molecular markers, with the aim we can use almost all the available sequences in the public dataset. However, *L. confundens* sequences that aligned with our ITS‐I locus were not available in public database, therefore no *L. confundens* sequences could be included in the concatenated dataset. Species with inadequate genetic resources needed to answer questions regarding evolutionary relatedness and genetic uniqueness are particularly problematic.

Published records of *L. confundens* to date are restricted to the eastern entrance of the Strait of Magellan and offshore, that is, on Burdwood Bank and Northern Scotia Ridge (Anosov et al., 2015; Sotelano et al., 2013), southwest Atlantic (Lovrich et al., 2002) and one record at Punta Arenas (53°S‐71°W, Tablado, 2021). The distribution range extension of this species to the other side of the continent would not be surprising, considering other examples of lithodids that have even been found in other hemispheres (Pérez‐Barros et al., 2020). This demonstrates the wide representativeness of the genus around the world, which evidences a potential for dispersal that we do not yet understand.

The delimitation of *Lithodes* IEUs on SEP should be extended to a more integrative taxonomy study that could include spermatic analysis, crossbreeding experiments, cytogenetic analysis, and comparisons of internal morphological traits less exposed to environmental factors (i.e., spermio‐taxonomy, gastric mills); this could help to identify the spatio‐temporal limits of the genetic lineages identified in our study.

In conclusion, we report evidence of two deeply divergent mitochondrial lineages of *Lithodes* from Valdivia (39°S) to Cape Horn (56°S) along the SEP with different zonal and meridional frequencies. Considerations for fishery management should recognize the mitochondrial genetic diversity observed here, with a special focus on those localities where this diversity is greatest. In this sense, it would be interesting to carry out phylogeographic analyses at a finer scale with other more variable molecular markers to define population boundaries for harvest management and recognize biodiversity hotspots in order to conserve them (e.g., Red King Crab in Alaska, Grant et al., 2014). However, before any consideration, the taxonomic status of *Lithodes* on SEP coasts should be re‐examined. The spatial complexity reported here, (i.e., different mitochondrial frequencies found in inner and open water areas with evidence of exchange of individuals among locations and latitudinal differentiation in haplotypes frequencies of each clade) underscores the urgency of understanding the evolutionary history of the *Lithodes* spp. across the SEP coasts.

## AUTHOR CONTRIBUTIONS

**Ramona Josefa Pinochet Sánchez:** Conceptualization (lead); data curation (lead); formal analysis (lead); funding acquisition (supporting); investigation (lead); methodology (equal); project administration (supporting); writing – original draft (lead). **Luis Miguel Pardo:** Conceptualization (supporting); funding acquisition (equal); investigation (supporting); methodology (supporting); project administration (equal); resources (equal); supervision (equal); validation (equal); writing – review and editing (equal). **Leyla Cárdenas Tavie:** Conceptualization (supporting); funding acquisition (equal); investigation (supporting); methodology (supporting); project administration (equal); resources (equal); supervision (equal); validation (equal); writing – review and editing (equal).

## CONFLICT OF INTEREST

None declared.

## Supporting information


Table S1
Table S1.2.
Figure S1.3
Figure S1.4

## Data Availability

The sequences of dataset were deposited in Genbank; COX‐I: ON807360‐ON807532; 28‐S: ON868924‐ON869058; ITS‐1: ON869067‐ON869217.

## References

[ece39143-bib-0001] Amaro, R. C. , Rodrigues, M. T. , Yonenaga‐Yassuda, Y. , & Carnaval, A. C. (2012). Demographic processes in the montane Atlantic rainforest: molecular and cytogenetic evidence from the endemic frog *Proceratophrys boiei* . Molecular Phylogenetics and Evolution, 62(3), 880–888. 22108674 10.1016/j.ympev.2011.11.004

[ece39143-bib-0002] Anosov, S. E. , Spiridonov, V. A. , Neretina, T. V. , Uryupova, E. F. , & Schepetov, D. (2015). King Crabs of the western Atlantic sector of Antarctic and adjacent areas: new records, molecular barcode data and distribution (Crustacea: Decapoda: Lithodidae). Polar Biology, 38(2), 231–249.

[ece39143-bib-0003] Balding, D. J. , & Nichols, R. A. (1995). A method for quantifying differentiation between populations at multi‐allelic loci and its implications for investigating identity and paternity. Genetica, 96(1–2), 3–12. 7607457 10.1007/BF01441146

[ece39143-bib-0004] Benson, D. A. , Clark, K. , Karsch‐Mizrachi, I. , Lipman, D. J. , Ostell, J. , & Sayers, E. W. (2014). GenBank. Nucleic Acids Research, 42(D1), D32–D37. 24217914 10.1093/nar/gkt1030 PMC3965104

[ece39143-bib-0005] Bickford, D. , Lohman, D. J. , Sodhi, N. S. , Ng, P. K. , Meier, R. , Winker, K. , Ingram, K. K. , & Das, I. (2007). Cryptic species as a window on diversity and conservation. Trends in Ecology & Evolution, 22(3), 148–155. 17129636 10.1016/j.tree.2006.11.004

[ece39143-bib-0006] Boschi, E. E. , & Gavio, M. A. (2005). On the distribution of decapod crustaceans from the Magellan Biogeographic Province and the Antarctic region. Scientia Marina, 69(S2), 195–200.

[ece39143-bib-0007] Bouckaert, R. , Vaughan, T. G. , Barido‐Sottani, J. , Duchêne, S. , Fourment, M. , Gavryushkina, A. , Heled, J. , Jones, G. , Kühnert, D. , de Maio, N. , Matschiner, M. , Mendes, F. K. , Müller, N. F. , Ogilvie, H. A. , du Plessis, L. , Popinga, A. , Rambaut, A. , Rasmussen, D. , Siveroni, I. , … Drummond, A. J. (2019). BEAST 2.5: An advanced software platform for Bayesian evolutionary analysis. PLoS Computational Biology, 15(4), e1006650. 30958812 10.1371/journal.pcbi.1006650 PMC6472827

[ece39143-bib-0008] Bozzeda, F. , Marín, S. L. , & Nahuelhual, L. (2019). An uncertainty‐based decision support tool to evaluate the southern king crab (*Lithodes santolla*) fishery in a scarce information context. Progress in Oceanography, 174, 64–71.

[ece39143-bib-0009] Bracken‐Grissom, H. D. , Cannon, M. E. , Cabezas, P. , Feldmann, R. M. , Schweitzer, C. E. , Ahyong, S. T. , Felder, D. L. , Lemaitre, R. , & Crandall, K. A. (2013). A comprehensive and integrative reconstruction of evolutionary history for Anomura (Crustacea: Decapoda). BMC Evolutionary Biology, 13(1), 1–29. 23786343 10.1186/1471-2148-13-128 PMC3708748

[ece39143-bib-0010] Carstens, B. C. , Pelletier, T. A. , Reid, N. M. , & Satler, J. D. (2013). How to fail at species delimitation. Molecular Ecology, 22(17), 4369–4383. 23855767 10.1111/mec.12413

[ece39143-bib-0011] Carvalho, G. R. , & Hauser, L. (1995). Molecular genetics and the stock concept in fisheries. In Molecular genetics in fisheries (pp. 55–79). Springer.

[ece39143-bib-0012] Carvalho, G. R. , & Nigmatullin, C. M. (1998). Stock structure analysis and species identification, FAO Fisheries Technical Paper. 199–232.

[ece39143-bib-0013] Chu, K. H. , Li, C. P. , & Ho, H. Y. (2001). The first internal transcribed spacer (ITS‐1) of ribosomal DNA as a molecular marker for phylogenetic and population analyses in Crustacea. Marine Biotechnology, 3(4), 355–361. 14961351 10.1007/s10126001-0014-5

[ece39143-bib-0014] Collins, R. A. , & Cruickshank, R. H. (2013). The seven deadly sins of DNA barcoding. Molecular Ecology Resources, 13(6), 969–975. 23280099 10.1111/1755-0998.12046

[ece39143-bib-0015] Crossley, S. A. (1986). Courtship‐sounds and behaviour in the four species of the *Drosophila bipectinata* complex. Animal Behaviour, 34(4), 1146–1159.

[ece39143-bib-0016] Cortese, G. , Gersonde, R. , Hillenbrand, C. D. , & Kuhn, G. (2004). Opal sedimentation shifts in the World Ocean over the last 15 Myr. Earth and Planetary Science Letters, 224(3–4), 509–527.

[ece39143-bib-0017] De Queiroz, K. (2007). Species concepts and species delimitation. Systematic Biology, 56(6), 879–886. 18027281 10.1080/10635150701701083

[ece39143-bib-0018] Denton, G. H. , Anderson, R. F. , Toggweiler, J. R. , Edwards, R. L. , Schaefer, J. M. , & Putnam, A. E. (2010). The last glacial termination. Science, 328(5986), 1652–1656. 20576882 10.1126/science.1184119

[ece39143-bib-0019] Diekmann, B. , Fälker, M. , & Kuhn, G. (2003). Environmental history of the south‐eastern South Atlantic since the Middle Miocene: Evidence from the sedimentological records of ODP Sites 1088 and 1092. Sedimentology, 50(3), 511–529.

[ece39143-bib-0020] Drummond, A. J. , Ho, S. Y. , Phillips, M. J. , & Rambaut, A. (2006). Relaxed phylogenetics and dating with confidence. PLoS Biology, 4(5), e88. 16683862 10.1371/journal.pbio.0040088 PMC1395354

[ece39143-bib-0021] Dudgeon, C. L. , Blower, D. C. , Broderick, D. , Giles, J. L. , Holmes, B. J. , Kashiwagi, T. , & Ovenden, J. R. (2012). A review of the application of molecular genetics for fisheries management and conservation of sharks and rays. Journal of Fish Biology, 80(5), 1789–1843. 22497408 10.1111/j.1095-8649.2012.03265.x

[ece39143-bib-0022] Dufresnes, C. , Brelsford, A. , Jeffries, D. L. , Mazepa, G. , Suchan, T. , Canestrelli, D. , Nicieza, A. , Fumagalli, L. , Dubey, S. , Martínez‐Solano, I. , & Litvinchuk, S. N. (2021). Mass of genes rather than master genes underlie the genomic architecture of amphibian speciation. Proceedings of the National Academy of Sciences, 118(36), e2103963118. 10.1073/pnas.2103963118 PMC8433553 34465621

[ece39143-bib-0023] Dupont, L. M. , & Leroy, S. A. (1999). Climatic changes in the Late Pliocene of NW Africa from a pollen record on an astronomically tuned timescale. In The Pliocene, time of change: American Association of Stratigraphic Palynologists Foundation (pp. 145–161). American Association of Stratigraphic Palynologists Foundation.

[ece39143-bib-0024] Feldmann, R. M. (1998). Paralomis debodeorum, a new species of decapod crustacean from the Miocene of New Zealand: first notice of the Lithodidae in the fossil record. New Zealand Journal of Geology and Geophysics, 41(1), 35–38.

[ece39143-bib-0025] Folmer, O. , Black, M. , Hoeh, W. , Lutz, R. , & Vrijenhoek, R. (1994). DNA primers for amplification of mitochondrial cytochrome c oxidase subunit I from diverse metazoan invertebrates. Molecular Marine Biology and Biotechnology, 3, 294–299. 7881515

[ece39143-bib-0026] Fong, P. P. , & Garthwaite, R. L. (1994). Allozyme electrophoretic analysis of the Hediste limnicola—H. diversicolor—H. japonica species complex (Polychaeta: Nereididae). Marine Biology, 118(3), 463–470.

[ece39143-bib-0027] Francuski, L. , Ludoški, J. , Vujić, A. , & Milankov, V. (2011). Phenotypic evidence for hidden biodiversity in the *Merodon aureus* group (Diptera, Syrphidae) on the Balkan Peninsula: conservation implication. Journal of Insect Conservation, 15(3), 379–388.

[ece39143-bib-0028] Fraser, C. I. , Nikula, R. , Ruzzante, D. E. , & Waters, J. M. (2012). Poleward bound: biological impacts of Southern Hemisphere glaciation. Trends in Ecology & Evolution, 27(8), 462–471. 22658874 10.1016/j.tree.2012.04.011

[ece39143-bib-0029] González‐Wevar, C. A. , Hüne, M. , Cañete, J. I. , Mansilla, A. , Nakano, T. , & Poulin, E. (2012). Towards a model of postglacial biogeography in shallow marine species along the Patagonian Province: lessons from the limpet *Nacella magellanica* (Gmelin, 1791). BMC Evolutionary Biology, 12(1), 139. 22871029 10.1186/1471-2148-12-139 PMC3582430

[ece39143-bib-0030] González‐Wevar, C. A. , Saucède, T. , Morley, S. A. , Chown, S. L. , & Poulin, E. (2013). Extinction and recolonization of maritime Antarctica in the limpet *Nacella concinna* (Strebel, 1908) during the last glacial cycle: toward a model of Quaternary biogeography in shallow Antarctic invertebrates. Molecular Ecology, 22(20), 5221–5236. 24102937 10.1111/mec.12465

[ece39143-bib-0031] Grant, W. S. , Zelenina, D. A. , & Mugue, N. S. (2014). Phylogeography of red king crab: Implications for management and stock enhancement. In B. Stevens (Ed.), The King Crabs (pp. 47–72). CRC Press.

[ece39143-bib-0082] Hall, S. , & Thatje, S. (2018). Evolution through cold and deep waters: The molecular phylogeny of the Lithodidae (Crustacea: Decapoda). The Science of Nature, 105(3), 1–15. 10.1007/s00114-018-1544-2 PMC5829116 29488024

[ece39143-bib-0032] Hansen, J. , Sato, M. , Russell, G. , & Kharecha, P. (2013). Climate sensitivity, sea level and atmospheric carbon dioxide. Philosophical Transactions of the Royal Society A: Mathematical, Physical and Engineering Sciences, 371(2001), 20120294. 10.1098/rsta.2012.0294 PMC3785813 24043864

[ece39143-bib-0033] Hebert, P. D. , Penton, E. H. , Burns, J. M. , Janzen, D. H. , & Hallwachs, W. (2004). Ten species in one: DNA barcoding reveals cryptic species in the neotropical skipper butterfly *Astraptes fulgerator* . Proceedings of the National Academy of Sciences, 101(41), 14812–14817. 10.1073/pnas.0406166101 PMC522015 15465915

[ece39143-bib-0034] Herrera, S. , & Shank, T. M. (2016). RAD sequencing enables unprecedented phylogenetic resolution and objective species delimitation in recalcitrant divergent taxa. Molecular Phylogenetics and Evolution, 100, 70–79. 26993764 10.1016/j.ympev.2016.03.010

[ece39143-bib-0035] Hodell, D. A. , & Venz, K. (1992). Toward a high‐resolution stable isotopic record of the Southern Ocean during the Pliocene‐Pleistocene (4.8 to 0.8 Ma). The Antarctic Paleoenvironment: A Perspective on Global Change: Part One, 56, 265–310.

[ece39143-bib-0036] Jones, G. (2017). Algorithmic improvements to species delimitation and phylogeny estimation under the multispecies coalescent. Journal of Mathematical Biology, 74(1–2), 447–467. 27287395 10.1007/s00285-016-1034-0

[ece39143-bib-0084] Jukes, T. H. , & Cantor, C. R. (1969). Evolution of protein molecules. Mammalian Protein Metabolism, 3, 21–132.

[ece39143-bib-0037] Kearse, M. , Moir, R. , Wilson, A. , Stones‐Havas, S. , Cheung, M. , Sturrock, S. , Markowitz, S. , Duran, C. , & Thierer, T. (2012). Geneious Basic: an integrated and extendable desktop software platform for the organization and analysis of sequence data. Bioinformatics, 28(12), 1647–1649. 22543367 10.1093/bioinformatics/bts199 PMC3371832

[ece39143-bib-0038] Ketmaier, V. , Argano, R. , & Caccone, A. (2003). Phylogeography and molecular rates of subterranean aquatic stenasellid isopods with a peri‐Tyrrhenian distribution. Molecular Ecology, 12(2), 547–555. 12535105 10.1046/j.1365-294x.2003.01734.x

[ece39143-bib-0039] King, M. (2007). Bhattacharya plots. In Fisheries Biology, Assessment and Management (2nd ed., pp. 368–371). Blackwell Publishing Ltd..

[ece39143-bib-0040] Kumar, S. , Stecher, G. , Li, M. , Knyaz, C. , & Tamura, K. (2018). MEGA X: molecular evolutionary genetics analysis across computing platforms. Molecular Biology and Evolution, 35(6), 1547–1549. 29722887 10.1093/molbev/msy096 PMC5967553

[ece39143-bib-0041] Lefébure, T. , Douady, C. J. , Gouy, M. , & Gibert, J. (2006). Relationship between morphological taxonomy and molecular divergence within Crustacea: proposal of a molecular threshold to help species delimitation. Molecular Phylogenetics and Evolution, 40(2), 435–447. 16647275 10.1016/j.ympev.2006.03.014

[ece39143-bib-0042] Lim, G. S. , Balke, M. , & Meier, R. (2012). Determining species boundaries in a world full of rarity: singletons, species delimitation methods. Systematic Biology, 61(1), 165–169. 21482553 10.1093/sysbio/syr030

[ece39143-bib-0043] Lovrich, G. A. (1997). La pesquería mixta de las centollas *Lithodes santolla* y *Paralomis granulosa* (Anomura: Lithodidae) en Tierra del Fuego, Argentina. Investigaciones Marinas, 25, 41–57.

[ece39143-bib-0044] Lovrich, G. A. , Perroni, M. , Vinuesa, J. H. , Tapella, F. , Chizzini, A. , & Romero, M. C. (2002). Occurrence of *Lithodes confundens* (Decapoda: Anomura) in the intertidal of the Southwestern Atlantic. Journal of Crustacean Biology, 22(4), 894–902.

[ece39143-bib-0045] Lovrich, G. A. (2014). Lithodidae. In Invertebrados del Mar Argentino (pp. 241–261). Vazquez Mazzini.

[ece39143-bib-0046] Lovrich, G. A. , & Tapella, F. (2014). Southern king crabs. King crabs of the world: biology and fisheries management, 636.

[ece39143-bib-0047] Machordom, A. , & Macpherson, E. (2004). Rapid radiation and cryptic speciation in squat lobsters of the genus *Munida* (Crustacea, Decapoda) and related genera in the South West Pacific: molecular and morphological evidence. Molecular Phylogenetics and Evolution, 33(2), 259–279. 15336662 10.1016/j.ympev.2004.06.001

[ece39143-bib-0048] Marlow, J. R. , Lange, C. B. , Wefer, G. , & Rosell‐Melé, A. (2000). Upwelling intensification as part of the Pliocene‐Pleistocene climate transition. Science, 290(5500), 2288–2291. 11125138 10.1126/science.290.5500.2288

[ece39143-bib-0049] Mayden, R. L. (1997). A hierarchy of species concepts: the denouement in the saga of the species problem. In M. F. Claridge , H. A. Dawah , & M. R. Wilson (Eds.), Species: The units of diversity (pp. 381–423). Chapman & Hall.

[ece39143-bib-0050] Meyer, R. , Weis, A. , & Melzer, R. R. (2013). Decapoda of southern Chile: DNA barcoding and integrative taxonomy with focus on the genera *Acanthocyclus* and *Eurypodius* . Systematics and Biodiversity, 11(3), 389–404.

[ece39143-bib-0051] Miller, M. A. , Pfeiffer, W. , & Schwartz, T. (2010). Creación de la Pasarela Científica CIPRES para la inferencia de grandes árboles filogenéticos. las Actas del Taller de Entornos de Computing Gateway (GCE), 14, 1–8.

[ece39143-bib-0052] Molinet, C. , Olguín, A. , Gebauer, P. , Díaz, P. A. , Díaz, M. , Matamala, T. , Mora, P. , & Paschke, K. (2020). Upswing and expansion of the southern king crab (*Lithodes santolla*) fishery in Northwest Patagonia: Drivers, trends and opportunities for management. Regional Studies in Marine Science, 34, 101073.

[ece39143-bib-0053] Montecinos, A. , Broitman, B. R. , Faugeron, S. , Haye, P. A. , Tellier, F. , & Guillemin, M. L. (2012). Species replacement along a linear coastal habitat: phylogeography and speciation in the red alga *Mazzaella laminarioides* along the south east pacific. BMC Evolutionary Biology, 12(1), 1–17. 22731925 10.1186/1471-2148-12-97 PMC3483259

[ece39143-bib-0054] Moore, W. S. (1995). Inferring phylogenies from mtDNA variation: mitochondrial‐gene trees versus nuclear‐gene trees. Evolution, 49(4), 718–726. 28565131 10.1111/j.1558-5646.1995.tb02308.x

[ece39143-bib-0055] Nahuelhual, L. , Saavedra, G. , Blanco, G. , Wesselink, E. , Campos, G. , & Vergara, X. (2018). On super fishers and black capture: Images of illegal fishing in artisanal fisheries of southern Chile. Marine Policy, 95, 36–45.

[ece39143-bib-0056] Naim, D. M. , Nor, S. A. M. , & Mahboob, S. (2020). Reassessment of species distribution and occurrence of mud crab (*Scylla* spp., Portunidae) in Malaysia through morphological and molecular identification. Saudi Journal of Biological Sciences, 27(2), 643–652. 32210683 10.1016/j.sjbs.2019.11.030 PMC6997873

[ece39143-bib-0057] Negri, M. , Lemaitre, R. , & Mantelatto, F. L. (2014). Molecular and Morphological Resurrection of *Clibanarius Symmetricus*, a Cryptic Species Hiding Under the Name for the “Thinstripe” Hermit Crab *C. vittatus* (Decapoda: Anomura: Diogenidae). Journal of Crustacean Biology, 34(6), 848–861.

[ece39143-bib-0083] Noever, C. , & Glenner, H. (2018). The origin of king crabs: Hermit crab ancestry under the magnifying glass. Zoological Journal of the Linnean Society, 182(2), 300–318.

[ece39143-bib-0058] Ortiz, D. , Pekár, S. , Bilat, J. , & Alvarez, N. (2021). Poor performance of DNA barcoding and the impact of RAD loci filtering on the species delimitation of an Iberian ant‐eating spider. Molecular Phylogenetics and Evolution, 154, 106997. 33164854 10.1016/j.ympev.2020.106997

[ece39143-bib-0059] Pérez‐Barros, P. , Confalonieri, V. A. , Paschke, K. , & Lovrich, G. A. (2015). Incongruence between molecular and morphological characters in the southern king crabs *Lithodes santolla* and *Lithodes confundens* (Decapoda: Anomura). Polar Biology, 38(12), 2097–2107.

[ece39143-bib-0060] Pérez‐Barros, P. , Albano, M. , Diez, M. J. , & Lovrich, G. A. (2020). Pole to pole: the deep‐sea king crab *Lithodes couesi* (Decapoda: Lithodidae) in the Burdwood Bank, Southwestern Atlantic Ocean. Polar Biology, 43(1), 81–86.

[ece39143-bib-0061] Pons, J. , Barraclough, T. G. , Gomez‐Zurita, J. , Cardoso, A. , Duran, D. P. , Hazell, S. , Kamoun, S. , Sumlin, W. D. , & Vogler, A. P. (2006). Sequence‐based species delimitation for the DNA taxonomy of undescribed insects. Systematic Biology, 55(4), 595–609. 16967577 10.1080/10635150600852011

[ece39143-bib-0062] Puillandre, N. , Lambert, A. , Brouillet, S. , & Achaz, G. (2012). ABGD, Automatic Barcode Gap Discovery for primary species delimitation. Molecular Ecology, 21(8), 1864–1877. 21883587 10.1111/j.1365-294X.2011.05239.x

[ece39143-bib-0063] Rambaut, A. , Drummond, A. J. , Xie, D. , Baele, G. , & Suchard, M. A. (2018). Posterior summarization in Bayesian phylogenetics using Tracer 1.7. Systematic Biology, 67(5), 901–904. 29718447 10.1093/sysbio/syy032 PMC6101584

[ece39143-bib-0064] Reid, N. M. , & Carstens, B. C. (2012). Phylogenetic estimation error can decrease the accuracy of species delimitation: a Bayesian implementation of the general mixed Yule‐coalescent model. BMC Evolutionary Biology, 12(1), 196. 23031350 10.1186/1471-2148-12-196 PMC3503838

[ece39143-bib-0065] Retamal, M. A. , & Moyano, H. I. (2010). Zoogeography of Chilean marine and freshwater decapod crustaceans. Latin American Journal of Aquatic Research, 38(3), 302–328.

[ece39143-bib-0066] Retamal, M. A. (2012). Los Lithodidae Chilenos. Punta Arenas (Chile). Anales del Instituto de la Patagonia Serie Ciencias Naturales, 21, 111–129.

[ece39143-bib-0067] Rostami, K. , Peltier, W. R. , & Mangini, A. (2000). Quaternary marine terraces, sea‐level changes and uplift history of Patagonia, Argentina: comparisons with predictions of the ICE‐4G (VM2) model of the global process of glacial isostatic adjustment. Quaternary Science Reviews, 19(14–15), 1495–1525.

[ece39143-bib-0068] Salzburger, W. , Ewing, G. B. , & Von Haeseler, A. (2011). The performance of phylogenetic algorithms in estimating haplotype genealogies with migration. Molecular Ecology, 20(9), 1952–1963. 21457168 10.1111/j.1365-294X.2011.05066.x

[ece39143-bib-0069] Schubart, C. D. , Diesel, R. , & Hedges, S. B. (1998). Rapid evolution to terrestrial life in Jamaican crabs. Nature, 393(6683), 363–365.

[ece39143-bib-0070] Schweitzer, C. E. , & Feldmann, R. M. (2001). New Cretaceous and Tertiary decapod crustaceans from western North America. Bulletin of the Mizunami Fossil Museum, 28, 173–210.

[ece39143-bib-0081] SERNAPESCA . (2020). Anuario estadístico de pesca 2016‐2019. Servicio Nacional de Pesca y Acuicultura (SERNAPESCA), Ministerio de Economía, Fomento y Turismo, Gobierno de Chile. Retrieved from http://www.sernapesca.cl/informacion‐utilidad/anuarios‐estadisticos‐de‐pesca‐y‐acuicultura

[ece39143-bib-0071] Sierpe, J. & Sanhueza, A. (2003). Las Pesquerías de Magallanes. Caracterización del Sector Pequero XII Región de Magallanes y Antártica Chilena. Servicio Nacional de Pesca.

[ece39143-bib-0072] Sigman, D. M. , Jaccard, S. L. , & Haug, G. H. (2004). Polar ocean stratification in a cold climate. Nature, 428(6978), 59–63. 14999278 10.1038/nature02357

[ece39143-bib-0073] Sotelano, M. P. , Gowland‐Sainz, M. F. , Diez, M. J. , & Lovrich, G. A. (2013). Distribution of Lithodes confundens Macpherson, 1988 (Decapoda, Anomura) along the Atlantic continental shelf of southern South America. Crustaceana, 86(2), 246–252.

[ece39143-bib-0074] Sotelo, G. , Morán, P. , & Posada, D. (2009). Molecular phylogeny and biogeographic history of the European Maja spider crabs (Decapoda, Majidae). Molecular Phylogenetics and Evolution, 53(1), 314–319. 19460449 10.1016/j.ympev.2009.05.009

[ece39143-bib-0075] Tablado, A. (2021). Museo Argentino de Ciencias Naturales" Bernardino Rivadavia"(MACN). Invertebrates National Collection (MACNIn).

[ece39143-bib-0076] Vitalis, R. , Dawson, K. , & Boursot, P. (2001). Interpretation of variation across marker loci as evidence of selection. Genetics, 158(4), 1811–1823. 11514464 10.1093/genetics/158.4.1811 PMC1461744

[ece39143-bib-0077] Waples, R. S. , Punt, A. E. , & Cope, J. M. (2008). Integrating genetic data into management of marine resources: how can we do it better? Fish and Fisheries, 9(4), 423–449.

[ece39143-bib-0078] Warnke, D. A. , Allen, C. P. , Muller, D. W. , Hodell, D. A. , & Brunner, C. A. (1992). Miocene‐Pliocene Antarctic glacial evolution: A synthesis of ice‐rafted debris, stable isotope, and planktonic foraminiferal indicators, ODP Leg 114. The Antarctic Paleoenvironment: A Perspective on Global Change: Part One, 56, 311–326.

[ece39143-bib-0079] Werding, B. , & Hiller, A. (2017). Description of a new species of Pachycheles (Decapoda, Anomura, Porcellanidae) from the southern Caribbean Sea. Crustaceana, 90(7–10), 1279–1288.

[ece39143-bib-0080] Xu, J. , Chan, T. Y. , Tsang, L. M. , & Chu, K. H. (2009). Phylogeography of the mitten crab Eriocheir sensu stricto in East Asia: Pleistocene isolation, population expansion and secondary contact. Molecular Phylogenetics and Evolution, 52(1), 45–56. 19236929 10.1016/j.ympev.2009.02.007

