# Supplementary material for: Assessing diversity of King Crab Lithodes spp. in the south‐eastern pacific using phylogeny and molecular species delimitation methods
Source: Ecol Evol. 2022 Jul 31;12(8):e9143. doi: 10.1002/ece3.9143 (PMC9339758; doi:10.1002/ece3.9143)
Supplement: Supplementary file 1 — Table S1 Table S1.2. Figure S1.3 Figure S1.4 [file ECE3-12-e9143-s001.docx]

Supplementary information

Table S1 Table with information about molecular markers and sequence length.

| Primer | Base Pairs | Sequence (5' --> 3') |
| --- | --- | --- |
| LCO1490 | 537 | GGTCAACAAATCATAAAGATATTGG |
| HC02198 | 537 | TAAACTTCAGGGTGACCAAAAAATCA |
| ITS1_Chu_F | 551 | CACACCGCCCGTCGCTACTA |
| ITS1_Chu_R | 551 | ATTTAGCTGCGGTCTTCATC |
| ITS4497L_F | 530 | GTTCCGGCCGCCTCTACT |
| 28S1605L_R | 530 | AATGCTTTAGCGCCATACCATTTTA |

Table S1.2. Taxa included in this study with GenBank accession numbers of the molecular marker.

| Family | Species | Accession number | | |
| --- | --- | --- | --- | --- |
|  |  | COX-I | ITS1 | 28S |
| Lithodidae | *Lithodes confundens* | HM020900 | - | - |
|  |  | HM020901 |  |  |
|  |  | KC196535 |  |  |
|  |  | KC196536 |  |  |
|  |  | KC196537 |  |  |
|  |  | KC196538 |  |  |
|  |  | KM887440 |  |  |
|  |  | KM887441 |  |  |
|  |  | KM887493 |  |  |
| Lithodidae | *Lithodes santolla* | HM020897 | HM021009 | AY596100 |
|  |  | HM020902 |  |  |
|  |  | KM887460 |  |  |
|  |  | KM887467 |  |  |
|  |  | KM887487 |  |  |
|  |  | KM887490 |  |  |
|  |  | KM887492 |  |  |
|  |  | KM887497 |  |  |
|  |  | HM020897 |  |  |
|  |  | HM020902 |  |  |
| Lithodidae | *Lithodes ferox* | KY426276 | HM021015 | HM020856 |
| Lithodidae | *Lithodes couesi* | DQ882086 | - | - |
| Lithodidae | *Lithodes aequispinus* | AF425308 | - | - |
| Lithodidae | *Lithodes nintokuae* | AB375135 | - | - |
| Lithodidae | *Paralomis sp.* | HM020934 | - | - |
| Hapalogastridae | *Hapalogaster mertensii* | KY426276 | - | - |
| Paguridae | *Pagurus brevidactylus* | MF490058 | - | - |


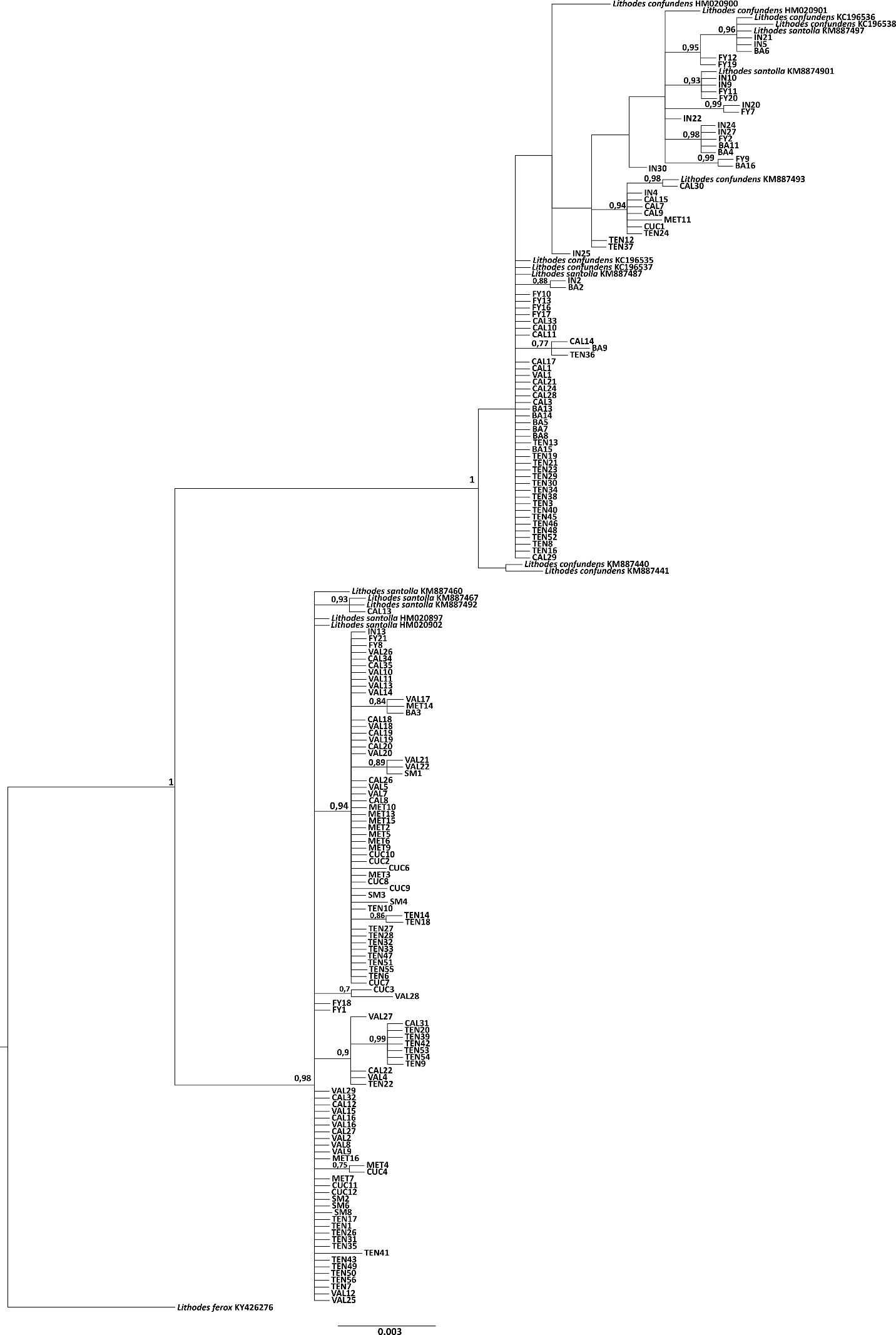


Figure S1.3 Phylogenetic reconstruction with Mrbayes of COX-I sequences from *Lithodes* sp. samples collected along 9 localities in ESP coast. Values above branches on the Bayes (BY) tree indicates the posterior probabilities of nodes of interest.


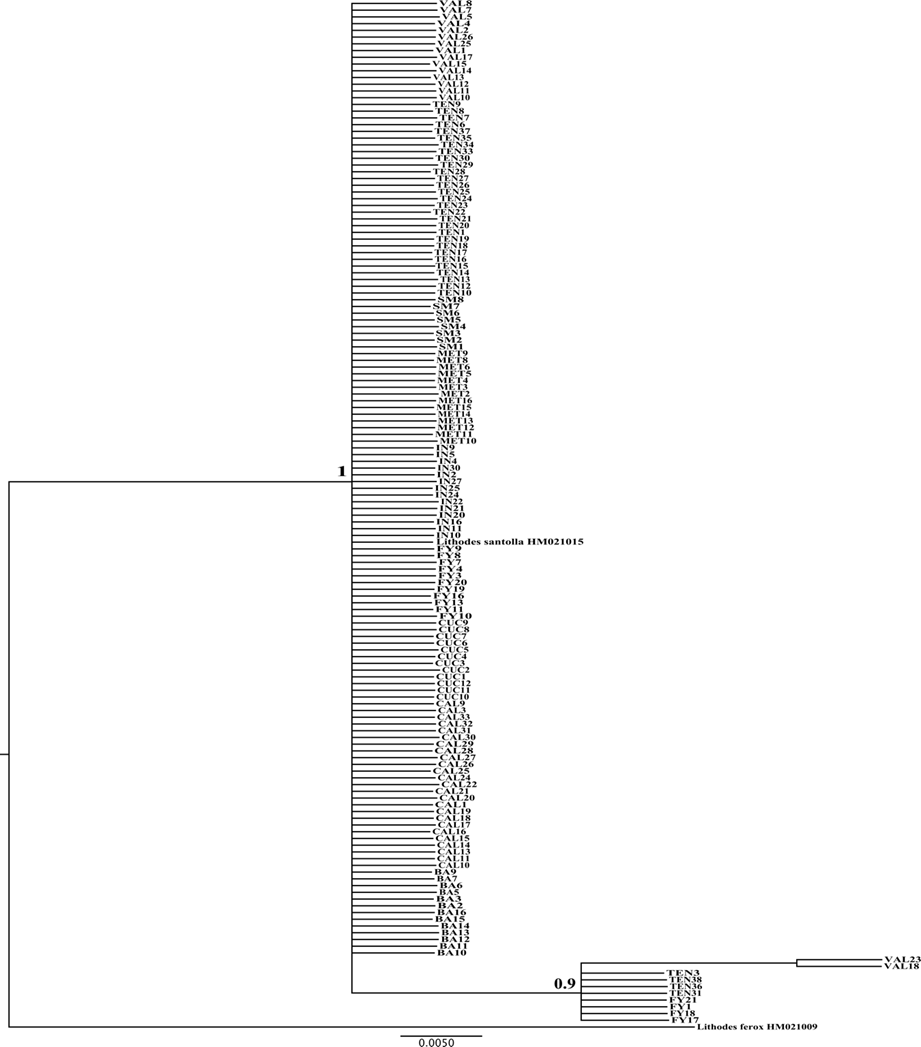


Figure S1.4 Phylogenetic reconstruction with MrBayes of ITS-1 sequences from *Lithodes* sp. samples collected along 9 localities in ESP coast. Values above branches on the Bayes (BY) tree indicates the posterior probabilities of nodes of interest.
